# Site selection and prediction of urban emergency shelter based on VGAE-RF model

**DOI:** 10.1038/s41598-024-64031-6

**Published:** 2024-06-22

**Authors:** Yong Wang, Yaoyao Han, An Luo, Shenghua Xu, Jian Chen, Wangwang Liu

**Affiliations:** 1 School of Geomatics, Anhui University of Science and Technology, Huainan, 232001 China; 2 Research Center of Geospatial Big Data Application, Chinese Academy of Surveying and Mapping, Beijing, 100830 China; 3 School of Geomatics and Marine Information, Jiangsu Ocean University, Lianyungang, 222002 China

**Keywords:** Emergency shelter site selection, Spatial correlation analysis, Variational graph auto-encoders, Feature fusion, Multi-source spatial data, Environmental social sciences, Natural hazards, Engineering

## Abstract

As urban development accelerates and natural disasters occur more frequently, the urgency of developing effective emergency shelter planning strategies intensifies. The shelter location selection method under the traditional multi-criteria decision-making framework suffers from issues such as strong subjectivity and insufficient data support. Artificial intelligence offers a robust data-driven approach for site selection; however, many methods neglect the spatial relationships of site selection targets within geographical space. This paper introduces an emergency shelter site selection model that combines a variational graph autoencoder (VGAE) with a random forest (RF), namely VGAE-RF. In the constructed urban spatial topological graph, based on network geographic information, this model captures both the latent features of geographic unit coupling and integrates explicit and latent features to forecast the likelihood of emergency shelters in the construction area. This study takes Beijing, China, as the experimental area and evaluates the reliability of different model methods using a confusion matrix, Receiver Operating Characteristic (ROC) curve, and Imbalance Index of spatial distribution as evaluation indicators. The experimental results indicate that the proposed VGAE-RF model method, which considers spatial semantic associations, displays the best reliability.

## Introduction

As urbanization progresses, the spatial structure of cities exhibits multi-centric characteristics, manifested in the densification and verticalization of construction, as well as the concentration and diversification of the population. Emergency shelters are gaining importance as critical urban facilities for responding to emergencies^1^. Yet, relative to the rapid expansion and complex evolution of cities, the planning and construction of these shelters frequently fall behind.

The site selection method for emergency shelters under the traditional multi-criteria decision-making framework first identifies candidate locations. Then, it evaluates their layout suitability based on some predetermined criteria^2,3^. However, these methods heavily rely on the subjective experiences of decision-makers, simplifying the high-dimensional and unstructured characteristics of an area. At the same time, the analysis process lacks big data support, resulting in biased location selection of emergency shelters. With smart city development, the surge in multi-source, heterogeneous urban spatial data offers rich data support for the site selection method of urban emergency shelters. The openness of urban spatial data, combined with internet geographical information acquisition, fusion, and analytical mining technology, opens new avenues in emergency shelter site selection research^4–6^. In contrast to traditional methods, artificial intelligence approaches driven by multi-source data can more effectively address existing limitations^7–9^. Some studies use an end-to-end analysis, employing data such as building outlines, user access trajectories, and urban points of interest to develop complex site selection models, applicable in commerce, healthcare, and transportation^10,11^. However, most multi-source data-driven approaches at this stage often treat candidate points or geographic units as isolated, overlooking the spatial network's proximity information in complex geography^12,13^. In recent years, some scholars have begun introducing Graph neural networks (GNNs) to deal with geographical spatial relationships and achieved certain results. However, most methods have been focused on predetermined positions in specific scenarios to build spatial graph convolution models or predict the attractiveness of establishments like stores and medical facilities based on user visit patterns or public transportation services^14–16^.

Consider that the location of shelters is not only intricately linked to urban structure, social dynamics, and geography, but also that residents' perception and visitation rates differ from those of other facilities. Existing methods of building spatial attribute graphs using GCNs may lead to inconsistent spatial attributes and insufficient spatial correlation analysis. This study aims to examine the limitations of existing site selection methods in processing spatial relationships and features. This paper establishes an urban spatial topological graph with consistent spatial meaning by simulating the service capabilities of emergency shelters, thereby closely linking discrete geographical units. Building upon, a novel method for city emergency shelter site selection is proposed, which integrates a variational graph auto-encoders (VGAE) and random forest (RF), referred to as VGAE-RF., and Beijing, China, is taken as experimental area. In addition, we introduce benchmark models such as support vector machines (SVM), K-nearest neighbors (KNN), and multi-layer perceptrons (MLP) for comparison of site selection prediction results before and after feature fusion to validate the feasibility and rationality of the proposed method.

This paper attempt to develop a new objective method for selecting urban emergency shelters from the perspective of artificial intelligence (AI) to assist city planning departments in making scientifically sound and rational decisions. The rest of this article is structured as follows: “Related research” outlines the research methodologies pertinent to emergency shelter location selection. “Methodology” details the method introduced in this study. “Experiment analysis” conducts a comparative analysis of the experimental results to verify the effectiveness of this method. “Conclusion and future work” summarizes the research characteristics of this article and looks forward to future research work.

## Related research

Site selection is widely regarded as a process of multi-criteria decision analysis. Researchers have developed various methods to meet diverse decision-making needs, broadly categorizing them into decision-maker experience-based models and AI-based predictive approaches^17,18^. (1) Experience-based site selection models typically begin by establishing a range of candidate points. Experts then define decision-making criteria based on site selection requirements and combined expertise. Geographic data are subsequently incorporated into a GIS analysis framework to identify the optimal location^19^. Methodologies in this category include the analytical hierarchy process (AHP)^20,21^, multiple attribute decision-making (MADM) methods^22,23^, multi-objective decision-making (MODM) methods^24,25^, and multi-criteria decision analysis (MCDA), among others^2,26,27^. For example, Trivedi et al. proposed a mixed multi-objective decision model employing the fuzzy hierarchical analysis method and the goal planning method to enhance post-disaster recovery engineering efficiency. This model aims to address the site selection and relocation issues of emergency shelters^28^. Considering the dynamic demands of shelters, Li et al. introduced a novel hierarchical planning model using a swapping allocation scheme to reduce evacuation distance and congestion in shelters, thereby identifying an emergency shelter that accommodates more disaster-affected individuals, lowers construction costs, and minimizes evacuation distance^29^. While these methods effectively utilize limited data for local site selection problems, they heavily depend on subjective rules set by decision-makers. Moreover, in the face of complex social and geographical environments, the massive volume of heterogeneous data renders these subjective methods unsustainable. (2) Artificial intelligence learning approaches utilize predictive methods such as random forest (RF), support vector machine (SVM), and multi-layer perceptron (MLP) classifiers to resolve optimal site selection problems^30–36^. Lu et al. integrated neural network regression prediction with the MCDM model to forecast hotel deployment locations using taxi GPS data^37^. Similarly, Huang et al. conducted planning research on SexyTea business outlet arrangements, employing the random forest model on a 100 m scale grid by merging multi-source spatial data from Changsha city^38^. These approaches enhance data processing efficiency, broaden the scope of site selection, and reduce inaccuracies in site selection results that are typically due to subjective biases. However, these methods reveal their inherent limitations. In geographic environments characterized by spatial correlation and heterogeneity, these approaches treat the target of site selection as an isolated entity, which is clearly not optimal^39^.

In site selection research that emphasizes spatial relationships, researchers have analyzed the interactions among various locations on a map and developed a geospatial graph convolutional neural network (GCN) based on the public transportation system. This network predicts the attractiveness of different store locations within a community^40^. However, constructing the GCN model necessitates avoiding node overlaps to prevent data leakage, which requires the consideration of geographical locations in dataset division. This ensures that nodes in close geographical proximity are separated. Such an approach might compromise graph semantics due to data segmentation. Additionally, these AI-based location selection methods are rarely applied to the study of urban emergency shelters. Consequently, by employing multiple sources of spatial data from the Internet, the VGAE-RF model has been developed as an unsupervised method for selecting sites for emergency shelters. This model not only addresses the limitations of subjective methods but also accounts for potential correlations in urban spaces.

## Methodology

**Figure 1:**
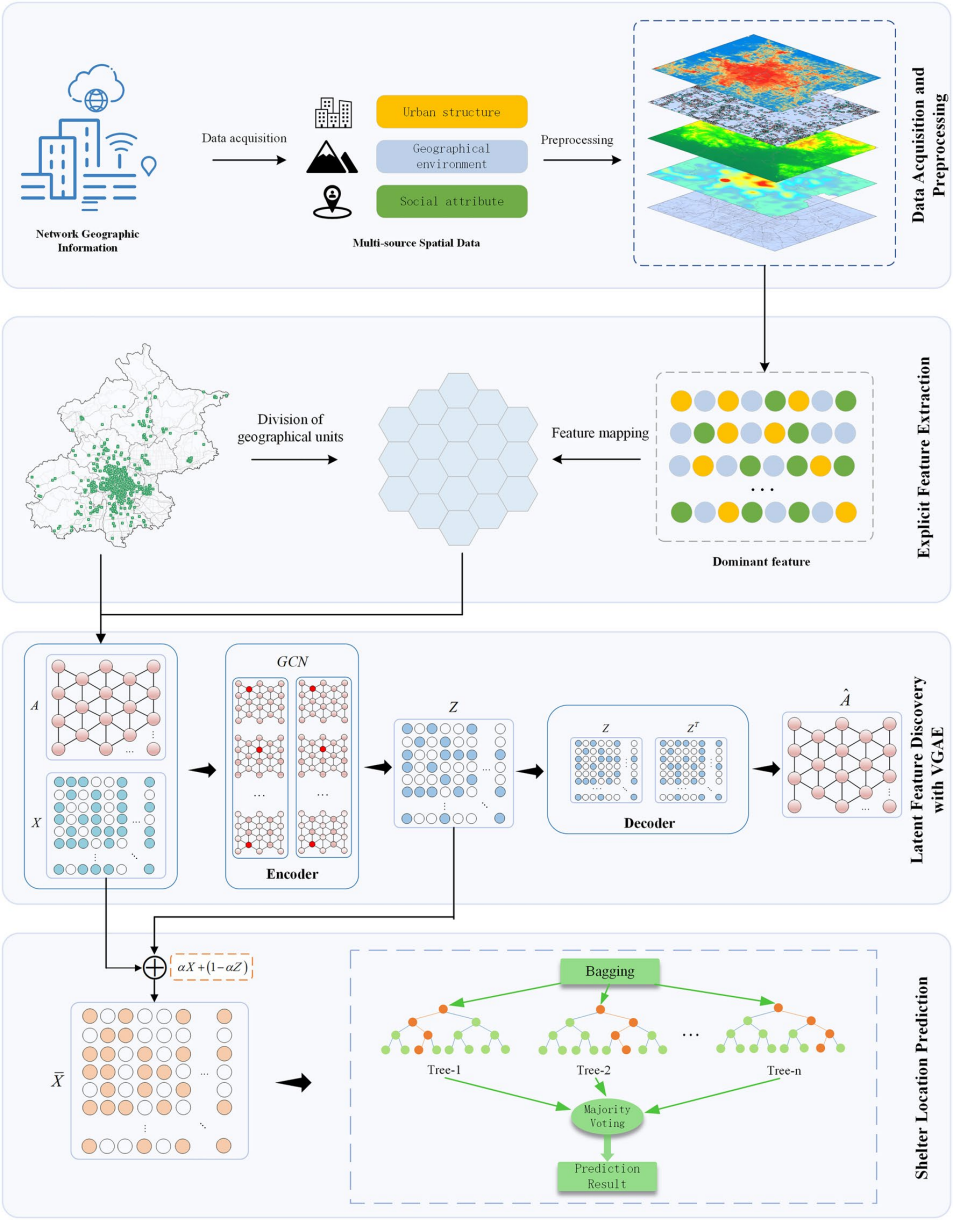
Overall methodological flowchart.

The key steps outlined in this article for selecting sites for urban emergency shelters comprise data acquisition and preprocessing, partitioning into grid units, extraction of explicit features, mining of latent features, and prediction of emergency shelter locations. This comprehensive framework is illustrated in Fig. 1.

### Data acquisition and preprocessing

The evolution of smart cities has led to an increase in geographical data available on the Internet, including basic geographical information, text tables, map images, and other formats. These urban spatial data have large volumes, diverse types, and disparate structures. This paper lays an essential foundation for analyzing and constructing the inherent multidimensional spatial attributes of emergency shelters^6,41^.

#### Data acquisition

This article employs search engines and web crawling technologies to gather information pertinent to the selection of emergency refuge sites from diverse sources on the Internet. These datasets include DEM data, POI data, locations of emergency shelters, population grids, road networks, and administrative divisions. Distinct strategies for data collection are applied based on the different types and structures of data features. (1) For instance, structured data like POI data typically exist as map service content, necessitating the utilization of Python scripts for batch downloading from open map platforms^42^. (2) Data published in the form of semi-structured data packets are obtained through engine searches and manual judgment collection. These data are frequently made available on platforms such as the Geospatial Data Cloud^43^ and the National Qinghai-Tibetan Plateau Scientific Data Center^44^, among others. (3) For unstructured statistical information, such as the Seventh National Population Census data and attribute information from shelters, crawler technology is used to retrieve and then further process this information into semi-structured information to supplement or verify structured geographic information.

#### Consistency processing

On the Internet, the spatial references and coordinate coding of multi-source heterogeneous geographical data vary, leading to issues such as missing or redundant data. This results in inconsistent expressions of geographical entities in terms of their geometry, attributes, and spatial relationships^45,46^. Therefore, prior to feature building, preprocessing of this multi-source heterogeneous data is essential. Initially, operations like data cleaning, coordinate transformation, geometric correction, and redundancy detection are conducted on the raw heterogeneous data collected online. These steps ensure the consistency of geographical data expression and lay the groundwork for data fusion and extraction. The treatment of spatial relationship consistency includes methods like mutual correction of population census data and grid data, road network topology repair, and alignment of emergency shelter positions. Finally, the reliability, completeness, and accuracy of the processed data, along with data quality, are validated and evaluated. The data units are then partitioned using the study area boundaries, facilitating the site selection of shelters at a fine scale.

### Space grid unit division

**Figure 2:**
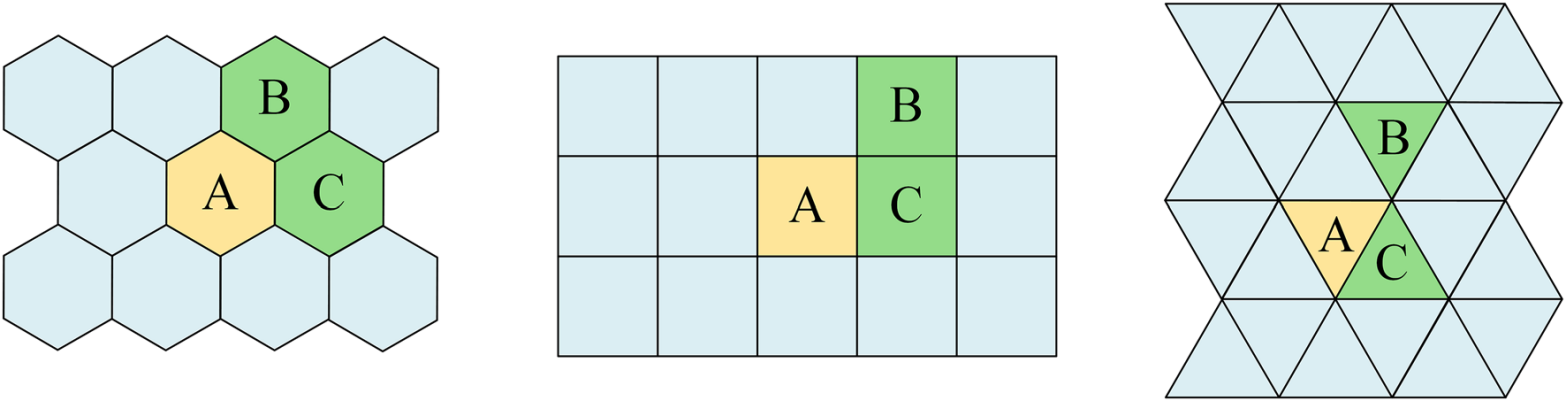
The connectivity paradox. In squares and triangles, it is impossible to determine whether there is a connection relationship between A, B, and C that has consistent spatial meaning. Regular hexagons have unique adjacencies determined only by their boundaries, so no such ambiguity exists^48^.

Three main types of regular polygon partition methods exist in plane space: triangles, squares, and hexagons^47,48^. Compared with a regular hexagon, using triangles and squares for determining adjacency relationships can lead to issues associated with the connectivity paradox (Fig. 2). Recently, hexagonal grids have gained popularity due to their consistent spatial implications of adjacency and higher adjacency density^49^. Specifically, the regular hexagon method offers several significant advantages in geographic spatial analysis: firstly, it provides uniform adjacency, effectively eliminating ambiguity in determining the adjacency of grid units^49^; secondly, it has a high perimeter-to-area ratio, which more efficiently reduces edge interference and its associated orientation and sampling biases^50^; thirdly, the distances from all edge points to the center point are uniform, enabling a more accurate representation of the multidirectional and multidimensional properties of spatial service range^51,52^. This study capitalizes on these benefits by adopting the regular hexagon as the basic unit for subdividing the spatial grid and utilizing its definite neighborhood relationships to construct the spatial topological graph.

To determine the scale of the grid units, this study investigated the time and walking distance required for residents in disaster cases to reach shelters; relevant domestic and foreign literature and construction standards were used, and 500 m was ultimately selected as the division scale^53,54^. The interior of the regular hexagonal grid at this scale is regarded as uniform, with the characteristic factor of each subdivision unit linked to its corresponding unique identifier (ID). This association is beneficial for precise spatial analysis.

### Explicit feature extraction

Site selection for urban emergency shelters is intricately connected with aspects like the urban geographic environment and population density. Based on contemporary research and analysis of multi-source data, this paper classifies the factors influencing urban emergency shelter selection into three critical dimensions: urban structure, geographic environment, and social attributes^26–29^. These dimensions include a range of data types and representations. In this section, various methodologies are utilized to extract explicit features from these dimensions.

#### Urban structure

1. Road network density

The density of the road network serves as a key indicator for assessing regional traffic accessibility. Typically, a higher road network density indicates more available routes for traffic diversion during passenger surges, reflecting the potential evacuation capabilities of urban community residents in disaster situations. In this paper, we start with four types of roads—urban main roads, urban secondary roads, urban branch roads, and elevated expressways—and comprehensively calculate the density of the road network. The process is represented by the ratio of the total length of roads within the geographical grid unit to the area of that grid unit.

1 $$D_{i} = \frac{{\sum\nolimits_{j = 1}^{4} {l_{ij} } }}{S}$$

2 $$D = [D_{1} ,D_{2} , \ldots ,D_{i} , \ldots D_{n} ]^{T} (1 \le i \le n)$$

Here, $D_{i}$ denotes the unnormalized road network density in the $i$ geographical grid unit, $l_{ij}$ denotes the length of the $j$ type of road in the unit, and $S$ the area of the geographic grid unit. $D$ is the column vector composed of $D_{i}$ values of all units. Next, the road network density undergoes linear normalization:

3 $$X_{{{\text{Road}}}} = \frac{{D - D_{\min } }}{{D_{\max } - D_{\min } }}$$

Here, $D_{\min }$ and $D_{\max }$ represent the minimum and maximum values of $D$, respectively, and $X_{{{\text{Road}}}}$ represents the values of road network density in all grid units after linear normalization.

(2) Architectural features.

The density and height of buildings significantly indicate the complexity of evacuation and the risk associated with refuge during emergencies. In regions with a higher density of high-rise buildings, there tends to be lower resilience for refuge in those areas. To quantify these characteristics, we constructed a building attribute matrix $B = [ID,H,BN]$, where $ID$ denotes the identification number of the geographical grid unit. $H$ and $BN$ denote the total building height and the number of buildings within the grid unit, respectively.

In geographic grid units, the building density $BD_{i}$ can be determined by the number of buildings per unit area, while the average building height $AH_{i}$ is expressed by the ratio of the total height to the number of buildings. The calculation formulas are as follows:

4 $$BD_{i} = \frac{{B_{i3} }}{S}$$

5 $$AH_{i} = \frac{{B_{i2} }}{{B_{i3} }}$$

Here, $B_{i2}$ denotes the cumulative height of buildings in geographic unit $i$. $B_{i3}$ signifies the quantity of buildings in geographic grid unit $i$, and $S$ represents the area of the geographic grid unit.

To mitigate the impact of dimensional units, it is essential to apply linear normalization to the features, thereby mapping their values to a range between 0 and 1.

6 $$BD = [BD_{1} ,BD_{2} , \ldots ,BD_{i} , \ldots ,BD_{n} ]^{T} (1 \le i \le n)$$

7 $$AH = [AH_{1} ,AH_{2} , \ldots ,AH_{i} , \ldots ,AH_{n} ]^{T} (1 \le i \le n)$$

8 $$X_{Building} = [\frac{{BD_{i} - BD_{min} }}{{BD_{max} - BD_{min} }},\frac{{AH_{i} - AH_{min} }}{{AH_{max} - AH_{min} }}]$$

Here, $BD$ and $AH$ are column vectors representing the building density $BD_{i}$ and average building height $AH_{i}$ for geographic grid unit $i$, respectively. $X_{Building}$ denotes the linearly normalized building feature matrix. $BD_{min}$, $BD_{max}$, $AH_{min}$ and $AH_{max}$ are the minimum and maximum values of the vectors $BD$ and $AH$, respectively.

#### Geographic environment

In the planning of emergency shelters, geographic environmental factors like slope and elevation play crucial roles. The slope impacts the accessibility of the shelter, whereas the elevation is intimately connected to the shelter's resilience against natural disasters like floods and its overall safety and stability during emergencies. Consequently, this paper develops geographic environmental characteristics from four perspectives: average elevation, average slope, standard deviation of slope, and standard deviation of elevation. Based on preprocessed DEM data, the slope information $Slop_{i}$ and elevation information $H_{i}$ can be represented as follows:

9 $$Slop_{i} = [s_{1} ,s_{2} , \ldots ,s_{i} , \ldots s_{m} ] (1 \le i \le m)$$

10 $$H_{i} = [h_{1} ,h_{2} , \ldots ,h_{i} , \ldots h_{m} ] (1 \le i \le m)$$

where $m$ is the number of sampling points within the geographic grid unit and $s_{m}$ and $h_{m}$ represent the slope and altitude at each sampling point, respectively. Correspondingly, the average altitude $\overline{H}_{i}$ and mean slope $\overline{Slop}_{i}$ can be computed by the following formulas:

11 $$\overline{H}_{i} = \frac{1}{m}\sum\limits_{k = 1}^{m} {h_{k} }$$

12 $$\overline{Slop}_{i} = \frac{1}{m}\sum\limits_{k = 1}^{m} {s_{k} }$$

Furthermore, the standard deviation of the slope can indicate the richness of the terrain in a region, and the standard deviation of the elevation can indicate susceptibility to natural disasters such as floods and landslides. The standard deviation of slope $\sigma_{Si}$ and elevation $\sigma_{Hi}$ are calculated as follows:

13 $$\sigma_{Si} = \sqrt {\frac{1}{m - 1}\sum\limits_{k = 1}^{m} {(s_{k} - \overline{{Slop_{i} }} )^{2} } }$$

14 $$\sigma_{Hi} = \sqrt {\frac{1}{m - 1}\sum\limits_{k = 1}^{m} {(h_{k} - \overline{{H_{i} }} )^{2} } }$$

#### Social attributes

1. Population density

Residents, as the service objects of emergency shelters, especially in densely populated areas, are more likely to need to construct shelters. To swiftly and effectively evacuate and accommodate a large number of people during a disaster or emergency, this study utilizes the ratio of the total population of a geographic grid unit to its area, calculated using the corrected gridded population data from the 7th National Census. The formula used for this calculation is:

15 $$Pop_{i} = \frac{{PN_{i} }}{S}$$

Here, $Pop_{i}$ represents the population density in geographical grid cell $i$, $PN_{i}$ represents the total population in geographical grid cell $i$, and $S$ represents the area of the geographical cell.

(2) POI density

A point of interest (POI) is defined as a specific location of public interest within a certain spatial area, reflecting, to some extent, the pattern of population activities in the region. It serves as one of the factors influencing the selection of emergency refuge locations. However, due to the diverse types of POIs and variable data quality, it becomes necessary to process and selectively categorize POI types relevant to emergency shelters. This paper reclassifies POIs within geographical units according to the 'Land Use Status Classification Standards (GB/T 21010–2017)' and relevant emergency field literature^48,58,59^. The classification includes eight types: residential services, dining and commercial services, industrial services, government functions, companies and enterprises, hazardous facilities, key evacuation sites, and parks and green spaces. The details of this classification are presented in Table 1.

**Table 1:** POI reclassified information.

Label	POI type	Content	Number
L1	Residential services	Residential buildings, Dormitories, Hotels, Hostels, Guest houses, etc	137,108
L2	Dining and commercial services	Fast food outlets, Restaurants, Supermarkets, Shopping centers, etc	394,370
L3	Industrial services	Factories, Mining companies, Metallurgical chemistry, Car repair, etc	19,184
L4	Government functions	Government agencies, Social groups, Industrial and commercial tax bureaus, Public inspection law institutions, etc	97,365
L5	Companies and enterprises	Companies, Agriculture and forestry horticulture, Parks, Office buildings, Advertising decoration, etc	97,905
L6	Parks and green spaces	Sports venues, Parks, Golf courses, Public squares, etc	5,606
L7	Hazardous facilities	Gas stations, Charging stations, Factory buildings, Warehouses, etc	20,818
L8	Key evacuation site	Hospitals, Schools, Train stations, Libraries, Museums, etc	52,908

Using the reclassified POIs, the density of each POI type is calculated by determining the ratio of the number of facilities in each geographical grid unit to the area of that grid. The formula for this calculation is as follows:

16 $$M = [N_{L1} ,N_{L2} , \ldots ,N_{Lj} , \ldots N_{Ln} ] (1 \le j \le n)$$

17 $$PD_{ij} = \frac{{M_{ij} }}{S}$$

Here, $M$ is the matrix of the number of facilities of each POI type in each geographical grid unit, $L_{j}$ is the $j$ type of POI, and $N_{Lj}$ is the number of facilities of the $L_{j}$ type of POI in grid unit $i$. $PD_{ij}$ represents the density of $L_{j}$ type POI facilities in grid unit $i$, $M_{ij}$ is the number of $L_{j}$ type POI facilities in grid unit $i$, and S is the area of each grid unit. Finally, the density matrix $PD$ of various POI densities $PD_{ij}$ is mapped between 0 and 1 through linear normalization:

18 $$X_{poi} (i,j) = \frac{{PD_{ij} - PDmin_{j} }}{{PDmax_{j} - PDmin_{j} }}$$

In the above equation, $X_{poi} (i,j)$ represents the normalized type $L_{j}$ POI facility density in geographic grid unit $i$, while $PDmin_{j}$ and $PDmax_{j}$ signify the minimum and maximum values, respectively, in each column of the matrix PD.

### Hidden feature mining based on VGAE

The first law of geography asserts that all things are interrelated, with closer entities being more closely related than those further apart^59^. In the context of emergency shelter site selection, it is vital to consider not just the explicit features within geographical grid units but also the potential impact of neighboring units. Accordingly, this paper constructs a regional spatial topological graph to model the association relationships among discrete geographical units in space. For this purpose, a regional spatial topological graph $G = (V,E)$ is constructed to fit the association relationships among discrete geographical units in space. This graph is based on the internal explicit features of the grid unit and its adjacency relationship. In the spatial topological graph $G$, $V$ represents the set of nodes corresponding to each geographical unit, and $E$ is the set of edges, where an element $$e_{ij} = (\begin{array}{*{20}c} {v_{i} ,} & {v_{j} } \\ \end{array} )$$ indicates adjacency between nodes $v_{i}$ and $v_{j}$.

**Figure 3:**
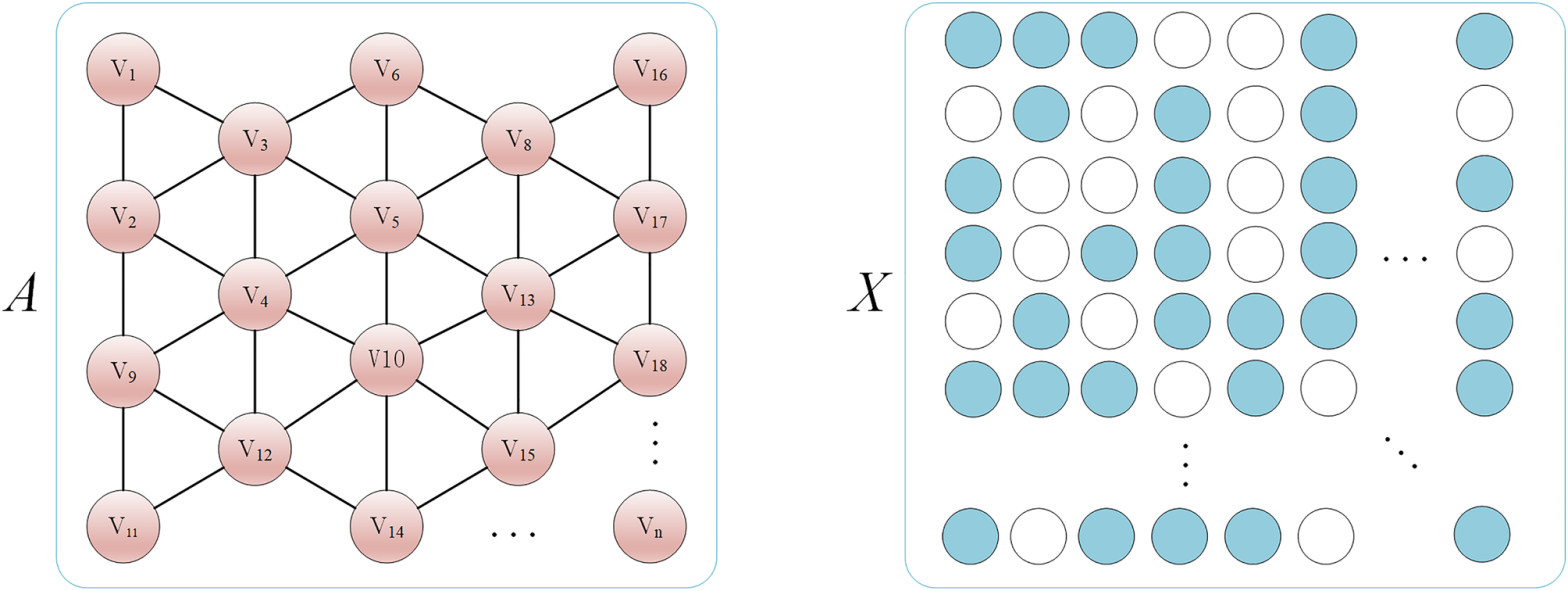
Graph adjacency matrix A and eigenmatrix X of the node.

As shown in Fig. 3, $v_{i} \in V$ denotes nodes in the graph, and $A$ represents the adjacency matrix of nodes. If $e_{ij} \in E$, then $a_{ij} { = 1}$ indicates that geographic units $i$ and $j$ are adjacent; otherwise, $a_{ij} { = 0}$. $X$ represents the feature matrix of nodes, where each node's attribute $x_{i}\in X$ represents the explicit feature of the corresponding geographical grid unit.

The spatial topological graph establishes connections among discrete geographic units^60,61^. Because of its ability to aggregate neighborhood information, the graph convolutional neural network (CGN) is capable of capturing the potential semantic features of nodes in a graph and has attracted the attention of many researchers^40^. Nevertheless, GCN could disrupt the overall semantics of the original graph during semi-supervised learning tasks, posing challenges in accurately capturing latent spatial patterns within the graph^62^. Therefore, we introduce variational graph autoencoders (VGAE). This model combines the advantages of graph autoencoders (GAE) and variational autoencoders (VAE) and can more deeply capture the intricate patterns in node representations^63^.

Specifically, the VGAE is a generative learning model tailored for processing graph data. It utilizes an encoder-decoder architecture to learn low-dimensional representations of nodes within a graph and subsequently reconstructs the graph's adjacency matrix. The encoder segment incorporates a two-layer graph convolutional network structure to process the explicit nodal features $X$ in a spatial topological graph. This process yields the mean and variance of the latent vector representation required. The operation of GCN can be articulated using the following formula:

19 $$\hat{A} = I + D^{{ - \frac{1}{2}}} AD^{{ - \frac{1}{2}}}$$

20 $$GCN\left( {X,A} \right) = \hat{A}Relu\left( {\hat{A}XW_{0} } \right)W_{1}$$

**Figure 4:**
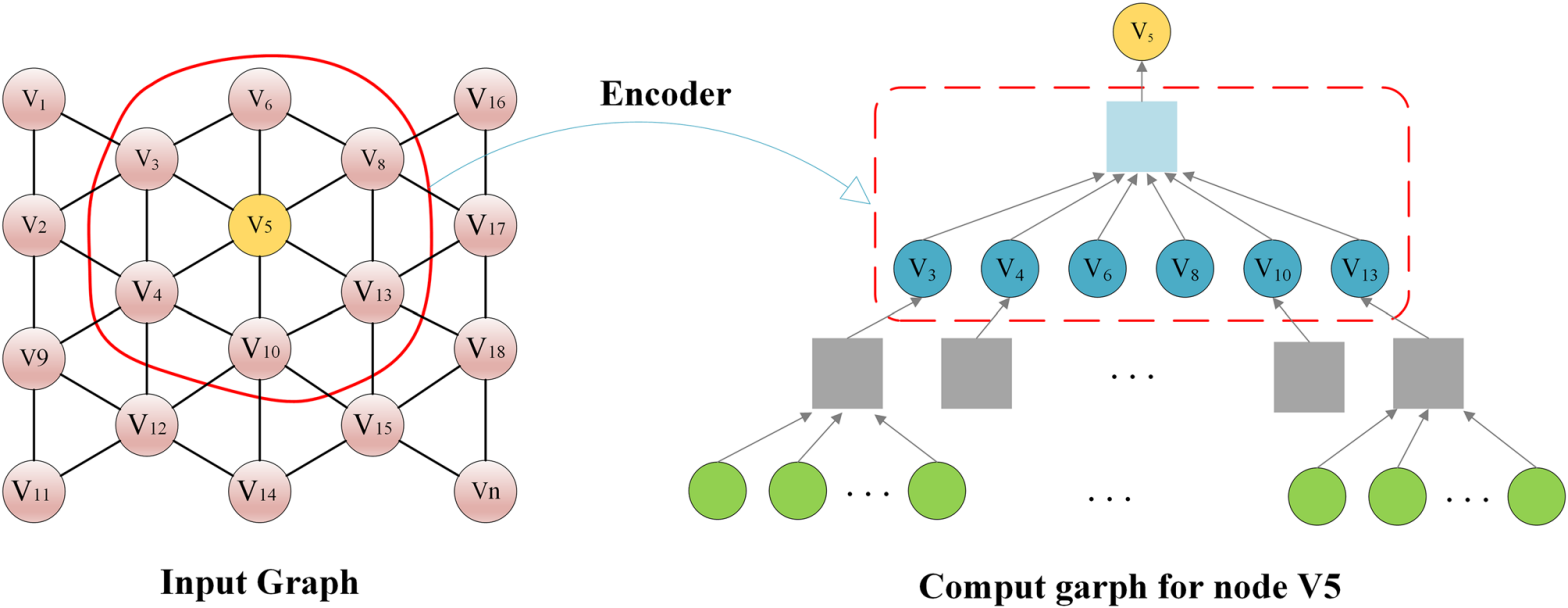
Autoinformatic aggregate neighborhood information.

Here, $\hat{A}$ is the normalized form of the adjacency matrix $A$. $I$ is the identity matrix, which represents connections to the node itself. $D$ is the degree matrix, denoting the number of edges connected to each node. $W_{0}$ and $W_{1}$ are the weight parameters. As shown in Fig. 4, the two-layer GCN structure effectively aggregates second-order neighborhood information, thereby helping individuals learn the latent spatial representation of the target geographical unit.

In the convolution process, the mean vector $\mu$ and the log covariance matrix $\log \sigma^{2}$ of the multidimensional Gaussian distribution can be learned from the second layer GCN of the encoder. The computation formulas are as follows:

21 $$\mu = GCN_{\mu } (X,A) = AH^{(1)} W_{\mu }^{(1)}$$

22 $$\log \sigma^{2} = GCN_{\sigma } (X,A) = AH^{(1)} W_{{\log \sigma^{2} }}^{(1)}$$

Here, $H^{(1)}$ represents the output of the first-layer graph convolution, $W_{\mu }^{(1)}$ and $W_{{\log \sigma^{2} }}^{(1)}$ represent the corresponding weight matrices.

Unlike the conventional GCN method, which directly extracts latent features from input data, VGAE obtains latent features by sampling from a multidimensional Gaussian distribution, which can be calculated as follows:

23 $$q\left( {z_{i} |X,A} \right) = N\left( {z_{i} |\mu_{i} ,diag(\sigma_{i}^{2} )} \right)$$

24 $$q(Z|X,A) = \prod\limits_{i = 1}^{N} {q\left( {z_{i} |X,A} \right)}$$

Here, $q\left( {z_{i} |X,A} \right)$ represents the distribution of the latent representation $z_{i}$ of nodes in a graph, while $N\left( {z_{i} |\mu_{i} ,diag(\sigma_{i}^{2} )} \right)$ indicates that $z_{i}$ abides by a multivariate Gaussian distribution. $q(Z|X,A)$ signifies the joint distribution of the latent representation Z of the spatial topological graph.

The decoder's objective is to sample latent features within a low-dimensional latent space and reconstruct the spatial topological structure of the graph through inner product operations. The expressions are as follows:

25 $$p\left( {A_{ij} = 1|z_{i} ,z_{j} } \right) = \sigma \left( {z_{i}^{\rm T} z_{j} } \right)$$

26 $$p(A|Z) = \prod\limits_{i = 1}^{N} {\prod\limits_{j = 1}^{N} p } \left( {A_{ij} |z_{i} ,z_{j} } \right) \,$$

Here, $p\left( {A_{ij} = 1|z_{i} ,z_{j} } \right)$ represents the probability of an adjacency edge between two nodes. $p(A|Z)$ is the probability of the entire graph's adjacency matrix occurring. $z_{i}$ and $z_{j}$ represent the latent feature vectors of geographical grid units $i$ and $j$, respectively, and $\sigma ( \cdot )$ is the sigmoid activation function.

The loss function of VGAE consists of two parts. One is the weighted cross-entropy loss function, measuring the dissimilarity between the original graph and the reconstructed graph. This guarantees the reconstructed graph's proximity to the original one, ensuring accurate modeling and learning of the data structure. The other part enforces the consistency of the latent space by minimizing the KL divergence between the encoder's output distribution and the prior distribution. This loss function can be expressed as follows:

27 $${\mathcal{L}} = {\mathbb{E}}_{{q({\mathbf{Z}}|{\mathbf{X}},{\mathbf{A}})}} [\log p(A|Z)] - {\text{KL}}[q(Z|X,A)\left\| {p(Z)} \right.]$$

Here, ${\mathbb{E}}_{{q({\mathbf{Z}}|{\mathbf{X}},{\mathbf{A}})}} [\log p(A|Z)]$ represents the expected reconstruction error, that is, the accuracy of reconstructing the original graph structure $A$ when the implicit feature $Z$ is given. ${\text{KL}}[q(Z|X,A)\left\| {p(Z)} \right.]$ is the kl divergence, which measures the difference between the output distribution $q(Z|X,A)$ of the encoder and the prior potential distribution *p*(*Z*).

### Feature fusion and location prediction

#### Fusion of explicit and latent features

In the realm of research and the application of various deep learning algorithms, integrating features at different hierarchical levels is a crucial approach to improving downstream task performance^64–66^. In this paper, explicit features include more intuitive attributes like building density and hazard sources, but they are constrained by their specific scale. Hence, we endeavor to merge these two categories of feature attributes through weighted fusion, specifically by combining the explicit features of nodes in the spatial topological graph with the latent features extracted by VGAE. The fusion formula is as follows:

28 $$\overline{X} = \alpha X + (1 - \alpha )Z$$

Here, $\overline{X}$ represents the fused features, with $\alpha$ representing the weighting coefficient.

#### Location prediction

This article comprehensively takes into account factors such as feature dimensions and nonlinear data. After comparing and analyzing various classical machine learning models and methods, the random forest (RF) algorithm was chosen as the classifier for site selection prediction. RF is an ensemble model consisting of multiple decision trees, which demonstrates excellent accuracy and generalizability when addressing high-dimensional nonlinear classification problems^67^. It builds a set of base estimators through K rounds of iterations, where each round of training utilizes the bootstrap method to extract samples from the original dataset with replacement. The final prediction is derived from a collective vote among decision trees over multiple rounds:

29 $$H(x) = arg\mathop {\max }\limits_{y} \mathop \sum \limits_{k = 1}^{K} I(h_{k} (X) = y)$$

Here, $H(x)$ is the final classification result output by the model. $y$ represents the given target variable, i.e., the label of each geographical grid unit. $I( \cdot )$ is an indicator function employed to determine whether the output of each decision tree $h_{i} (X)$ is equal to the given target variable y. shows the process of explicit and latent feature fusion and location prediction.

**Figure 5:**
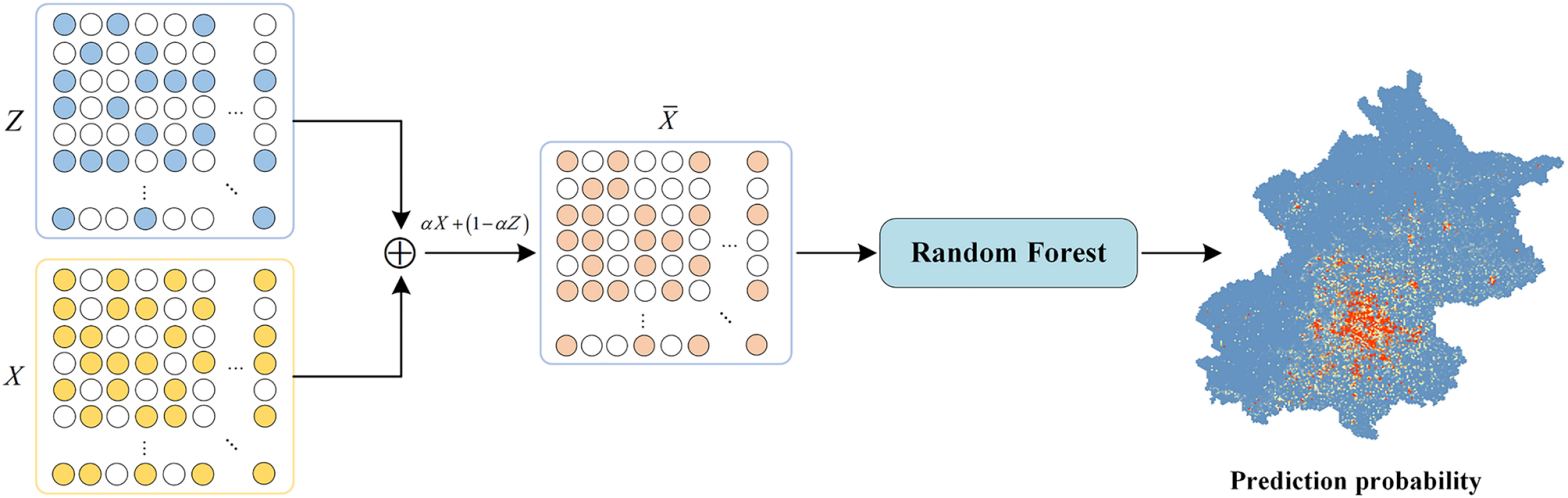
Explicit and latent feature fusion and location prediction. The map image was created by the authors using ArcGIS software version 10.8 (http://www.esri.com).

The construction process of the RF model is as follows: firstly, the emergency shelter site selection is simplified into a binary classification problem, where a geographical grid unit containing an emergency shelter is taken a dependent variable, and the fused features are taken as independent variables. Then, the relationship between and the indices of geographical grid units are used to construct the training dataset, by randomly selecting 70% of it as training data, and the remaining 30% along with out-of-bag (OOB) data as validation data. Finally, the pre-trained random forest model is employed for classification to obtain the probability distribution of emergency shelter locations on each grid (Fig. 5).

## Experiment analysis

This study selected Beijing, the capital of China, as the experimental area for the proposed methods. Beijing, as the political center of China, cultural center, international communication center, and technology innovation center, possesses remarkable authority and advancements in emergency management planning. In this section, we provide a comprehensive description of the data utilized in the experiment, the evaluation methods employed, and the parameter configurations. We then proceed to compare VGAE-RF with various models. Finally, by confirming the visualization results of the VGAE-RF model in the experimental area, we further discuss and demonstrate its effectiveness.

### Experimental data

During the experiment, the multi-source geospatial data we used were collected from open data collected on the Internet. The specific data acquisition times and sources are listed in Table 2 in detail to ensure the traceability and accuracy of the data.

**Table 2:** Data description and sources.

Type	Time	Sources	Description
POI	2021	Amap API data open interface (https://lbs.amap.com)	Call Amap API crawling through Python web crawler technology, a total of 1,239,707 pieces of data in 23 categories
Road network	2021	OpenStreetMap (https://www.openstreetmap.org)	Urban road network at all levels, used to calculate road network density
Population	2020	WorldPop (www.worldpop.org)	Spatial resolution is 3 radians (approximately 100 m at the equator)
DEM	2019	Geospatial Data Cloud (https://www.gscloud.cn)	The spatial resolution is 30 m, reflecting the topography of the study area
Building outline	2020	The National Tibetan Plateau Data Center (https://data.tpdc.ac.cn)	Rooftop vectorized data of 90 cities in China
Building height	2020	Zenodo (https://zenodo.org)	Building height data with nationwide coverage at 10-m resolution
Administrative division	2020	National Catalogue Service For Geographic Information (https://www.webmap.cn)	Characterizes the boundaries of Beijing's administrative divisions and is used to extract explicit features within the region
Statistical data	2020	The People's Government of Beijing Municipality (https://www.beijing.gov.cn)	Reflect the population distribution of each district and county
Shelter statistics	2022	Beijing Emergency Management Bureau (http://yjglj.beijing.gov.cn)	Contains attribute information of emergency shelters to supplement the deficiencies of POI data

### Evaluation criteria

For model reliability, on one hand, from the perspective of machine learning, traditional evaluation metrics such as the Kappa coefficient and accuracy overlook the posterior probability of the model. The confusion matrix provides detailed classification information of the model across different categories, enabling a more comprehensive assessment of the model's performance beyond merely focusing on overall prediction accuracy. Therefore, based on the confusion matrix, this paper selects precision, recall, and F1 score, and receiver operating characteristic (ROC) curve as performance metrics to assess for the model^68^. In the confusion matrix, true positives (TP), true negatives (TN), false positives (FP), and false negatives (FN) are defined as shown in Table 3.

**Table 3:** A 2 × 2 Confusion Matrix.

Total shelters = P + N		Predicted condition	
		Positive	Negative
Actual condition	Positive (P)	True positive (TP)	False negative (FN)
	Negative (N)	False positive (FP)	True negative (TN)

The calculation of the model's accuracy, recall rate, and F1 score is as follows:

30 $$\mathit {Precision} = \frac{TP}{{TP + FP}}$$

31 $$\mathit {Recall} = \frac{TP}{{TP + FN}}$$

32 $$F_{1} = \frac{2TP}{{2TP + FP + FN}}$$

where $TP$ is the number of true positives, $FP$ is the number of false positives, and $FN$ is the number of false negatives. Moreover, when considering the entire city, the relative scarcity of emergency shelters compared to other public service facilities results in class imbalances within the training samples. To address this, the receiver operating characteristic (ROC) curve and the area under the ROC curve (AUC) are employed. These metrics not only effectively assess the classifier's performance but also mitigate evaluation biases stemming from the uneven distribution of samples. The ROC curve illustrates the classifier's performance at various thresholds by plotting the true positive rate (TPR) against the false positive rate (FPR), and the AUC value represents the area beneath the ROC curve, ranging from 0 to 1. A higher AUC value indicates a superior model. The true positive rate (TPR) and false positive rate (FPR) can be calculated as follows:

33 $$TPR = \frac{TP}{{TP + FN}}$$

34 $$FPR = \frac{FP}{{FP + TN}}$$

On the other hand, considering the geographical spatial distribution pattern, the reliability of the model's spatial analysis capability is determined using the Imbalance Index. The Imbalance Index is an important indicator of the balance of the distribution of elements across different locations within the study area. In this paper, the Imbalance Index is utilized to quantify the balance of the model's site selection outcomes across various districts and counties, thereby revealing the fairness and reliability of the model's geographical spatial analysis. The calculation formula is as follows:

35 $$S = \frac{{\sum\nolimits_{i = 1}^{n} {Y_{i} } - 50(n + 1)}}{100n - 50(n + 1)}$$

where $S$ represents the Imbalance Index, ranging from 0 to 1. A higher value of $S$ indicates a more concentrated distribution, while a lower value of $S$ suggests a more balanced distribution.$n$ denotes the number of districts and counties in Beijing. $Y_{i}$ refers to the cumulative percentage of the i-th ranked district or county in descending order of the proportion of emergency shelters.

### Model parameters

The unsupervised learning characteristic of VGAE enables the avoidance of dataset division when mining latent features hidden within the urban spatial topological graph. This, in turn, mitigates the issue of incomplete learning of latent features due to data splitting. In this paper, in the encoder part of VGAE, the model takes node features as inputs and is set to train for a maximum of 3000 epochs, with early stopping when the validation loss has not seen improvement in the last 50 epochs. The list of hyperparameters is presented in Table 4.

**Table 4:** VGAE model hyperparameters.

Hyperparameters	VGAE
Hidden layer	16
Learning rate	0.05
Weight decay	5e−4
Dropout rate	0.00

**Figure 6:**
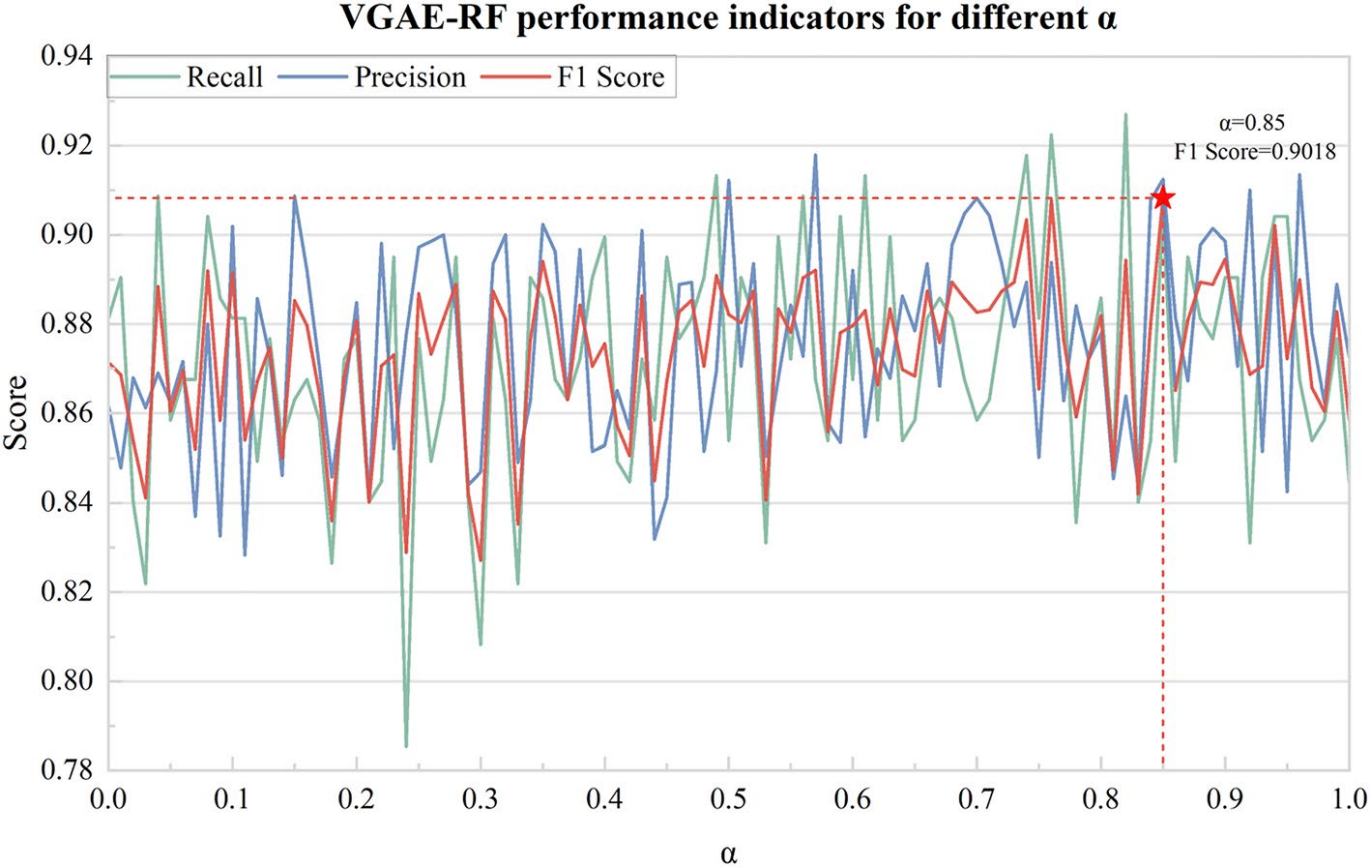
Variation curve of VGAE-RF performance (accuracy, recall, F1 score) for different fusion parameter α values.

To select the best feature fusion weight parameter, this study conducted predictive experiments by successively sampling the alpha from the interval 0 to 1 at a step size of 0.01. Figure 6 shows the evaluation results of different feature fusion weight parameters α in the VGAE-RF model. When α = 0.85, the F1 score (0.9018) is the best, and the model performance is optimal.

### Model reliability analysis

In this experiment, we employed tenfold cross-validation to evaluate the overall performance of the VGAE-RF model by calculating the average values of precision, recall, and $F_{1}$ score. To further validate the reliability of the proposed method, several baseline models were introduced for comparative analysis with VGAE-RF, including RF, SVM, KNN, and MLP. Subsequently, we replaced the original method's random forest base classifier with these baseline models to analyze the performance of the fused features under different base classifiers. Finally, the VGAE-RF-NF model, which does not fuse the original features, is compared with VGAE-RF. Table 5 presents the evaluation scores of the different models in these experiments.

**Table 5:** Comparison of evaluation scores of different models.

Compare objects	Precision	Recall	F1 score	Imbalance index
Existing shelters	**–**	**–**	**–**	0.4436
RF	0.8623	0.8467	0.8492	0.4642
SVM	0.8211	0.8604	0.8344	0.4667
KNN	0.8267	0.8493	0.8378	0.4635
MLP	0.8673	0.8950	0.8809	0.4582
VGAE-SVM	0.8528	0.8995	0.8756	0.4426
VGAE-KNN	0.8405	0.8904	0.8647	0.4463
VGAE-MLP	0.8722	**0.9041**	0.8879	0.4421
VGAE-RF-NF	0.8616	0.8813	0.8713	0.4573
**VGAE-RF**	**0.9112**	0.8904	**0.9018**	**0.4403**

Significant values are in bold.

The results demonstrated that the VGAE-RF model had the highest accuracy (0.9112) and highest score (0.9018), along with a relatively lower Imbalance Index (0.4403). Its recall rate is 0.8904, a value slightly lower than the highest recall score (0.9041) achieved by the VGAE-MLP model.

1. Among all methods, the reliability of non-neural network methods (RF, SVM, KNN) that directly predict without considering neighborhood unit coupling effects is the worst. This is because these simple models encounter challenges in learning complex feature relationships among geographical units. Once the region is subdivided into surfaces, the model's receptive field is limited to single-scale geographical units, making it unable to capture potential semantic relationships between these units.
2. In spatial prediction methods that incorporate explicit and implicit feature fusion using spatial topological graphs, there is a degree of performance improvement compared to that of their corresponding simple baseline models. This suggests that the latent influence of neighboring units is a nonnegligible factor, and VGAE effectively captures this latent spatial relationship. Simultaneously, the VGAE-RF model achieves the highest reliability, which can be attributed to the ensemble learning concept and the characteristics such as the random selection of feature subsets inherent in the final classifier, the Random Forest (RF). These features improve the robustness and generalization capability of the site selection process, providing better modeling capabilities for irregular decision boundaries.
3. When comparing the results before and after the fusion of explicit and implicit features, methods that do not fuse original features (VGAE-RF-NF) outperform non-neural network methods, but the fused VGAE-RF model exhibits even greater reliability. In essence, aggregating the features of second-order neighbor nodes through VGAE may lead to some loss of information about the original features. This loss is effectively addressed by feature fusion techniques.

**Figure 7:**
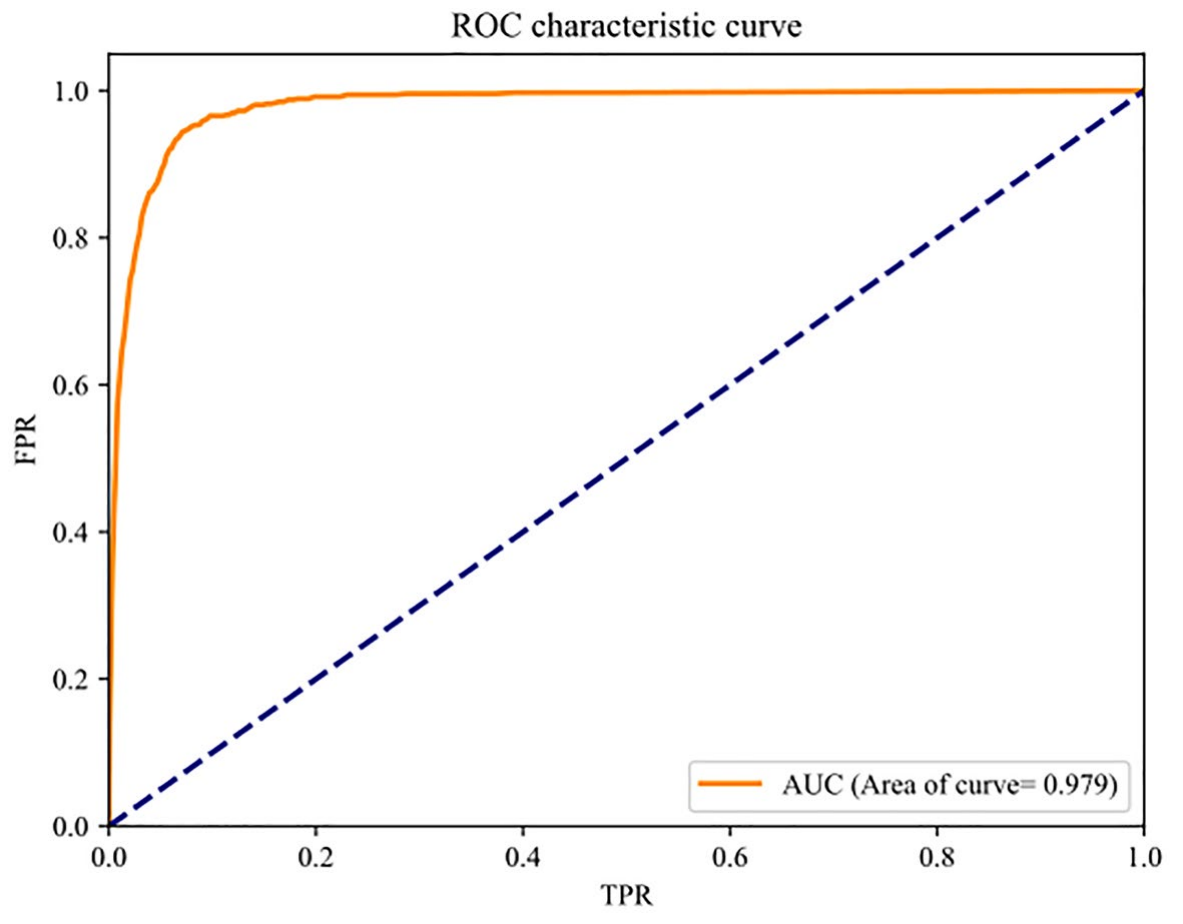
ROC curve and AUC values.

To further analyze the performance of the VGAE-RF model, we plotted the ROC curve (Fig. 7), and it can be seen that the curve exhibits a pronounced tilt toward the upper left quadrant, and the AUC area in this state was 0.979. This characteristic signifies the model's robust performance, even with a low false positive rate and a high true positive rate, highlighting its effectiveness in handling imbalanced datasets and its high precision and classification robustness.

### Results and discussion

In this subsection, we select the top 600 geographic grid units with the highest model recommendation ratings as potential candidates for future refuge construction based on population distribution, area, and existing emergency shelters in various regions of Beijing. Subsequently, we visually compare the performance of different models in terms of site selection suitability and analyze their differences in location selection. Additionally, we compare the site selection results predicted by the VGAE-RF model with the spatial layout of the current emergency shelters and explore the impact of various influencing factors on shelter location selection aiming to reveal the application potential and practical effects of VGAE-RF in shelter site selection.

#### Model site selection differences

**Figure 8:**
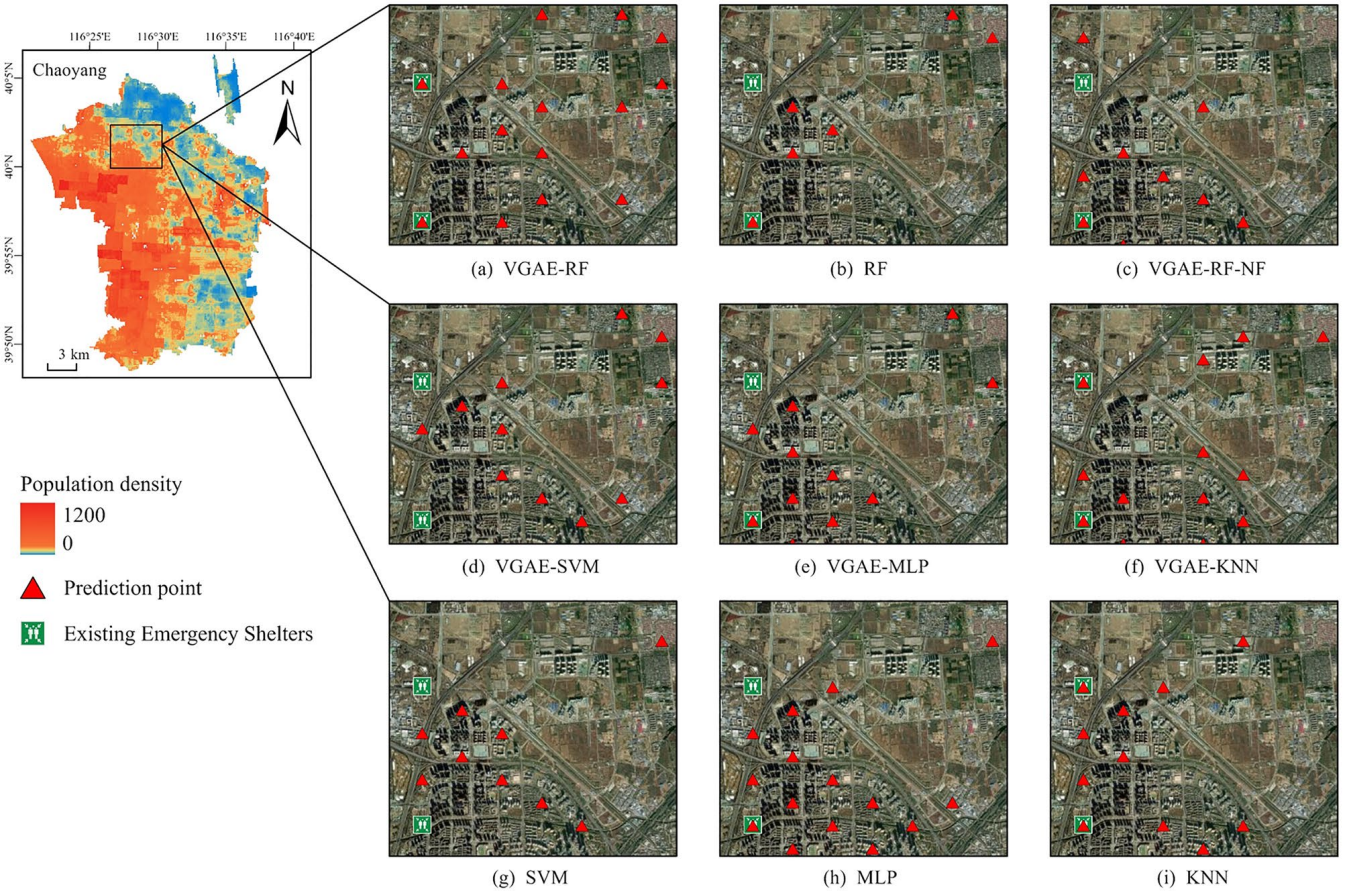
Visualization of site selection details for each model in the Laiguangying area, Chaoyang District. The map image was created by the authors using ArcGIS software version 10.8 (http://www.esri.com).

To further investigate the differences in model applications, this paper selects the Laiguangying region in the Chaoyang District, where spatial patterns have changed significantly in recent years, as a case study to intuitively compare the performance of different models in terms of site suitability (Fig. 8).

The results indicate that all models accurately identify regions with sparse populations, but they differ in pinpointing shelter locations. Among these models, models (RF, SVM, MLP, and KNN) that consider only the internal characteristics of geographical grid cells exhibit poor suitability for predicting point locations in this range—they fall short in identifying current emergency shelters and maintaining a balanced consideration for site arrangements. In contrast, models that consider the influence of neighboring units (VGAE-RF, VGAE-SVM, VGAE-KNN, and VGAE-MLP) strike a balance between ensuring the largest possible service range and avoiding hazardous areas, such as densely built-up high-rise areas.

#### Spatial prediction layout of VGAE-RF

**Figure 9:**
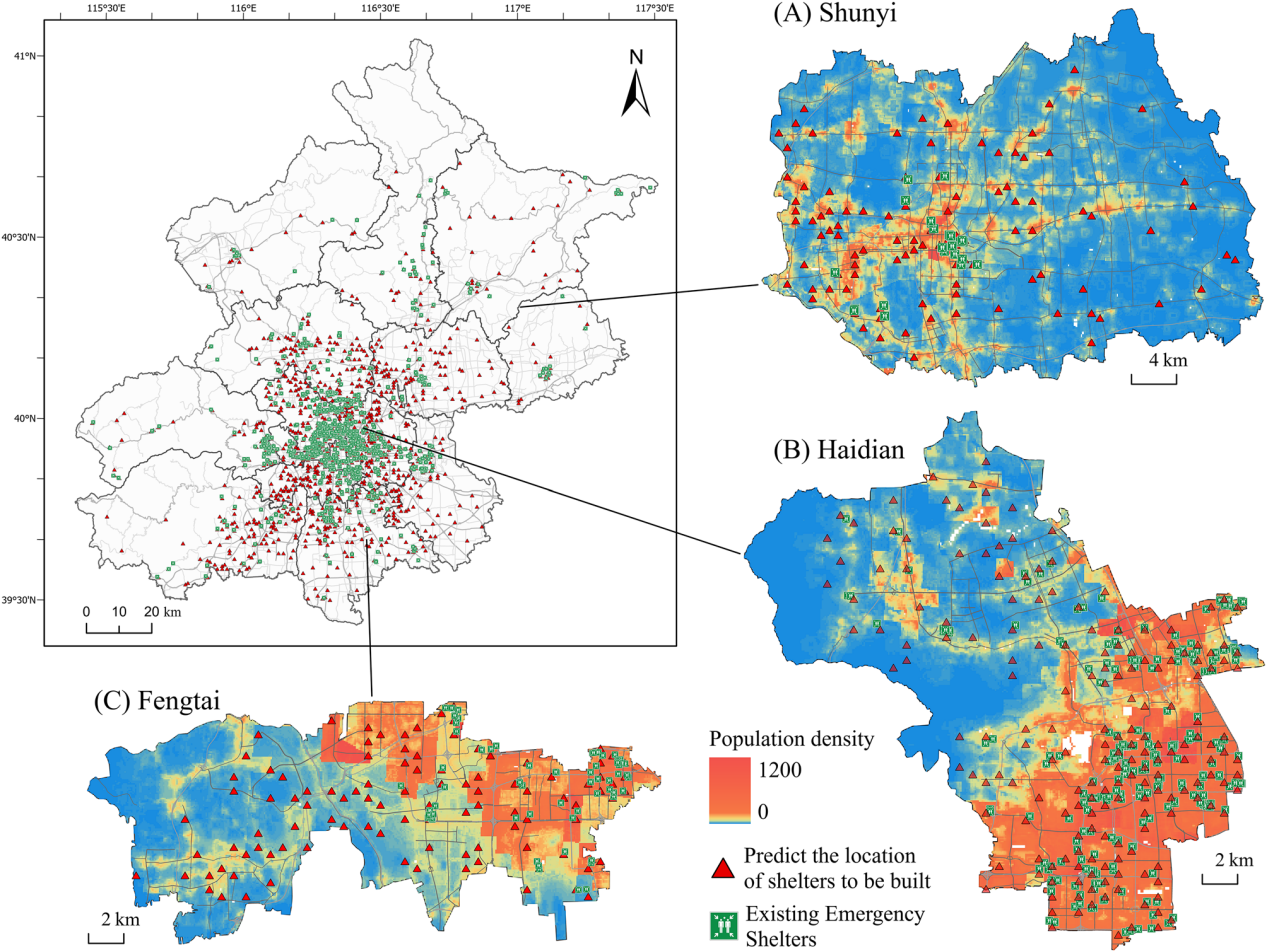
VGAE-RF refuge site selection results compared with those of existing shelters and combined with population density. Th e site selection details of (A) Shunyi District, (B) Haidian District, and (C) Fengtai District are displayed. The map image was created by the authors using ArcGIS software version 10.8 (http://www.esri.com).

We utilize the site selection results for (A) Shunyi District, (B) Haidian District, and (C) Fengtai District as examples, comparing the VGAE-RF site selection outcomes with the existing emergency evacuation shelters (Fig. 9).

1. From a spatial distribution perspective, VGAE-RF accurately identifies regions where refuges should be established but have not been yet. Furthermore, the hotspot areas for site selection closely align with population density, suggesting that more citizens can benefit from these shelters during emergencies.
2. Considering the urban development trends, Haidian and Fengtai districts are integral parts of the central city, while Shunyi District serves as a key area absorbing functions and population from the central city. These regions face challenges such as delayed construction and low site suitability for shelters. The model's prediction points in these areas are not only in proximity to key infrastructure, such as medical and transportation facilities but also distant from potential disaster-prone areas like gas stations, liquefied gas stations, and other high-risk facilities. In the long term, this approach contributes to enhancing urban disaster prevention resilience.

#### Effect of feature factors

**Figure 10:**
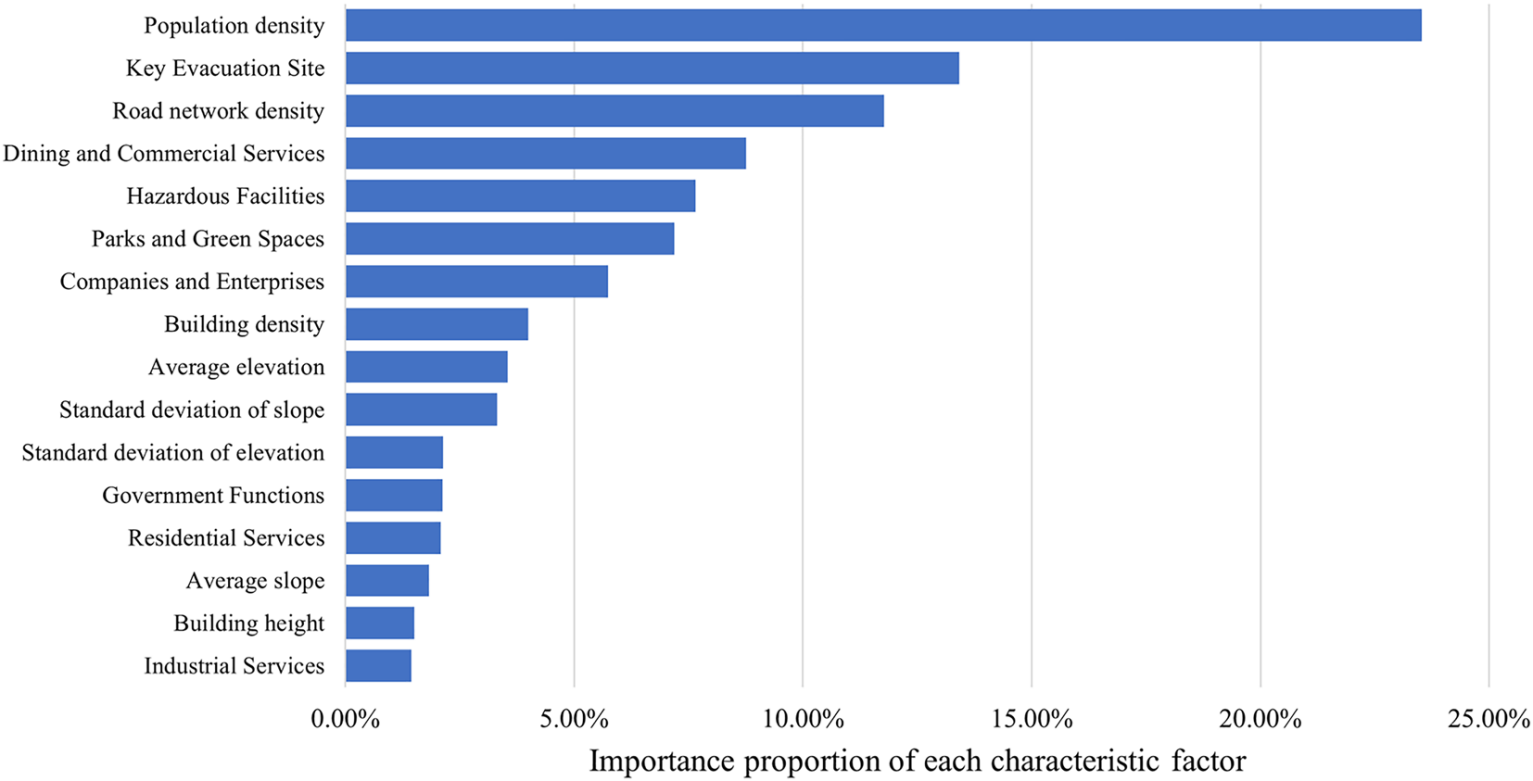
The importance of each index feature.

In the prediction process of the VGAE-RF model, we tried to use the Mean Decrease Gini (MDG) method to measure the importance of features and explore their impacts on the selection of refuge sites (Fig. 10).

The research indicates that among numerous factors affecting the selection of models, urban structure and social attributes play a vital role in the location selection of emergency shelters, which aligns with the conclusions drawn by Ashish Trivedi^26^ and Trivedi A^28^. Among them, the population density (23.52%) and urban road network density (11.77%) significantly affect the selection of shelters. According to the spatial location theory, areas with high population density can ensure that more people can quickly reach shelters in emergencies, essential for improving emergency response efficiency and reducing potential casualties. At the same time, the density of urban road networks directly affects the convenience and efficiency of transportation, especially during emergency evacuations. A high-density network can provide more route options, reduce traffic congestion, and accelerate evacuation speed. Given that the main urban area of Beijing is primarily on the plains, the impact of the geographical environment on the location of emergency shelters may not be prominent but remains an indispensable part of the risk assessment of the shelters. For instance, shelters in hilly or low-lying areas may become unsafe or unreachable due to topographical reasons during disasters such as earthquakes or floods.

## Conclusion and future work

In this study, we introduce a VGAE-RF site prediction method designed to extract and integrate potential urban spatial features and apply them to the site selection of emergency shelters. Using multi-source and heterogeneous networked urban spatial data, the VGAE-RF model can identify and aggregate both explicit and implicit features from urban spatial topological graph in geographically correlated and heterogeneous environments, leading to accurate site selection predictions. Our models give more accurate predictions than methods that do not consider neighborhood unit coupling effects, as demonstrated by the results. As highlighted in our experimental results, the latent semantic information within urban spaces plays a crucial role in optimizing the layout of emergency shelters. By utilizing this approach to shelter site selection, which takes spatial correlation into account, decision makers in emergency management can formulate more scientifically grounded and dependable strategies for urban shelter layout planning.

Based on previous research and discussions, our plan for the future work includes:

1. A more comprehensive set of influencing factors should be considered. Future research endeavors could investigate additional factors that might impact the site selection of emergency shelters, including vulnerable areas within cities, disaster-prone locations, and socioeconomic conditions. Such investigations aim to improve the predictive capacity and practical applicability of the model.
2. Improving the spatial semantic relationships in the urban spatial topological graph. This study created a topological graph of urban space characterized by consistent spatial relationships but lacking semantic associations. Therefore, additional associated relationships should be considered in the future to increase the rigor and reason of information transmission on maps.
3. Exploiting the advantages of multi-model fusion. In certain non-first-tier cities, the availability of multi-source spatial data is relatively limited, which constrains a single model's capability to make location selections in diverse areas. Consequently, harnessing the strengths of various models in discerning spatial features can enhance the model's adaptability.

## Data Availability

The datasets used or analyzed in the current study are described in “Experiment analysis” and are available from the corresponding author on reasonable request.
